# Abdominal Symptoms During the Febrile Phase Indicate Profound Innate Immune Responses in Dengue

**DOI:** 10.3390/biology15120960

**Published:** 2026-06-18

**Authors:** Huy Thanh Do, Thansita Bhunyakarnjanarat, Kanthaporn Dityen, Yadah Kaewopas, Niramol Thammachareonrach, Supaporn Paiboonkasarp, Thiranut Jaroonwitchawan, Siwaporn Boonyasuppayakorn, Wiwat Chancharoenthana, Asada Leelahavanichkul

**Affiliations:** 1 Doctor of Philosophy Program in Clinical Sciences (International Program), Faculty of Medicine, Chulalongkorn University, Bangkok 10330, Thailand; huythanhdo123@gmail.com; 2 Department of Microbiology, Faculty of Medicine, Chulalongkorn University, Bangkok 10330, Thailand; thansitadew@gmail.com (T.B.); kanthaporn.d@chula.ac.th (K.D.); yadah.k@chula.ac.th (Y.K.); ni.tham75@gmail.com (N.T.); supaporn.pa@chula.ac.th (S.P.); siwaporn.b@chula.ac.th (S.B.); 3 Center of Excellence in Translational Research on Immunology and Immune-Mediated Diseases (CETRII), Department of Microbiology, Faculty of Medicine, Chulalongkorn University, Bangkok 10330, Thailand; 4 Futuristic Science Research Center, School of Science, Walailak University, Thasala, Nakhon Si Thammarat 80160, Thailand; thiranut.ja@wu.ac.th; 5 Department of Clinical Tropical Medicine, Faculty of Tropical Medicine, Mahidol University, Bangkok 10400, Thailand; wiwat.cha@mahidol.ac.th; 6 Tropical Immunology and Translational Research Unit (TITRU), Department of Clinical Tropical Medicine, Faculty of Tropical Medicine, Mahidol University, Bangkok 10400, Thailand; 7 Division of Nephrology, Department of Medicine, Faculty of Medicine, Chulalongkorn University, Bangkok 10330, Thailand

**Keywords:** NETs, FGF-21, dengue, leaky gut, microbiome

## Abstract

Gastrointestinal symptoms (GI) (abdominal pain, vomiting, and diarrhea) during the febrile phase of dengue (less than 5 days from fever onset) might indicate prominent innate immune responses. Serum and feces samples from cases with GI symptoms versus those without GI symptoms (*n* = 20 per group) were analyzed. From these, only the neutrophil extracellular traps (NETs), serum fibroblast growth factor (FGF) 21, and fecal microbiome analyses, but not the routine parameters, endotoxemia, or serum cytokines, were higher in the GI cases than in the non-GI cases. From the in vitro experiments, both lipopolysaccharide (LPS) and the dengue virus (DENV) upregulated the FGF receptor 1 (*FGFR1*) and cytokines in hepatocytes (HepG2) and THP-1-differentiated macrophages. Meanwhile, LPS and DENV induced NETs in isolated neutrophils from healthy volunteers. Only the starvation protocol, but not LPS or DENV, enhanced supernatant FGF-21 from hepatocytes. Incubation of recombinant FGF-21 in LPS + DENV-activated cells (hepatocytes, macrophages, and neutrophils) attenuated inflammation, as determined by supernatant cytokines and NETs. Hence, abdominal symptoms in dengue during the febrile phase indicate prominent innate immune responses, as detected by NETs and FGF-21 (an acute-phase protein), implying significant hepatic stress with a possible counteracting anti-inflammation.

## Simple Summary

The innate immune responses during the early febrile phase of dengue might be able to determine disease severity during the critical phase. Here, we found that patients with digestive issues with fever exhibited higher levels of innate immune responses, as indicated by the neutrophil extracellular traps (NETs; web-like structures released by white blood cells to neutralize infection), increased serum fibroblast growth factor 21 (FGF-21; a protein indicating liver stress), and imbalances in gut bacteria. In the in vitro experiments, the dengue virus triggered inflammation (supernatant cytokines) and upregulated the FGF receptor in several cells (hepatocytes, macrophages, and neutrophils), while starvation induced FGF-21 production by hepatocytes. Incubation of recombinant FGF-21 reduced inflammation in these cells, suggesting that the liver produces FGF-21 as a natural counter response to reduce tissue damage. Hence, innate immunity is correlated with dengue severity, and monitoring NETs and FGF-21 in the febrile phase might identify high-risk patients. More studies on innate immunity in dengue would be interesting.

## 1. Introduction

Dengue, a significant mosquito-borne infection, demonstrates a wide range of symptoms, from mild fever to multiple organ failure, especially between the third and seventh days after the onset [1,2]. About four to five days after a mosquito bite, dengue begins with the viremia in the febrile phase (1 to 3 days after the fever onset), followed by the potentially hemoconcentration in the critical phase (3 to 7 day) and the recovery phase (after 7 days) [3]. The presence of warning signs (abdominal pain or tenderness, vomiting, fluid accumulation with ascites or pleural effusion, mucosal bleeding, lethargy or restlessness, liver enlargement, and hemoconcentration) indicates severe dengue that typically starts in the critical phase (1 to 2 days after the fever has subsided) [4]. Notably, 15–40% of patients with dengue have abdominal pain during the transition from the febrile to the critical phase (the non-febrile phase), and 60–70% of these patients develop severe disease [4]. While the importance of abdominal pain during the transition to the fever critical phase non-febrile phase is established as a dengue warning sign [5], the importance of abdominal pain during the febrile phase is still uncertain. Perhaps, the intestinal symptoms (abdominal pain, vomiting, and diarrhea) during the febrile phase might also be the parameters for the severe disease. While abdominal pain during the non-fever critical phase is well-known to be caused by plasma leakage, fluid accumulation, liver swelling, and organ inflammation from the adaptive immune responses (antibody-dependent enhancement and T cell-mediated cytokine storm) [5], the abdominal pain during the febrile phase might indicate the earlier prominent innate immune-mediated inflammation.

However, abdominal pain and vomiting are non-specific symptoms of fever from many causes, and hemoconcentration with thrombocytopenia is usually found in the non-fever critical phase [6,7,8]. Thus, the parameters of innate immunity (cytokines, neutrophil responses, and acute phase proteins) that indicate severe dengue during the febrile phase might be interesting to use together with these early intestinal symptoms. Regarding neutrophil responses during the febrile phase, neutrophilia in the early phase of dengue (days 1–5) before the leucopenia is common [9], and neutrophil extracellular traps (NETs; the web-like decondensed chromatin and histones with antimicrobial proteins) are associated with plasma leakage from endothelial glycocalyx degradation during dengue infection [7]. Hence, the neutrophil-associated parameters in the febrile phase are interesting. For acute-phase proteins (APPs) (mostly liver-associated parameters), there are several APPs that might be correlated with liver swelling in dengue [10]. Although the routine liver-associated biomarkers, including alanine transaminase (ALT) and C-reactive protein (CRP) [11,12], are not consistently associated with dengue disease severity, fibroblast growth factors (FGFs) are novel APPs produced from a stressed livers that might be correlated with dengue [13,14,15,16]. There are 22 isotypes of FGFs that induce tyrosine kinase through the FGF receptor types 1 to 4 with paracrine or autocrine activity for tissue repair and several metabolic activities [17]. Among these, FGF-21 is mainly synthesized by the liver to regulate several immune cells [18] with limited data on it in dengue. Thus, abdominal symptoms during the febrile phase of dengue might be associated with elevated NETs (a representative of the neutrophil responses) or FGF-21 (an interesting APP), and possibly indicate a severe innate immune responses. Because of the anti-inflammatory properties of FGF-21 [19], the elevated FGF-21 during infection might be a counter response against to severe inflammation.

Not only the innate immunity parameters, but also gut dysbiosis (alteration of gut microbiota that is harmful to the host) in dengue due to stress and the direct enterocyte infection [20], might be demonstrated during the febrile phase dengue by abdominal symptoms. Because (i) the dengue-induced viremia occurs during the febrile phase; (ii) the direct infection of enterocytes with dengue virus has been reported in mice [21]; and (iii) fecal dysbiosis might be quickly changed even within 24 h after some infections [22], gut dysbiosis might start during the early phase of dengue. Gut dysbiosis might also be correlated with the translocation of microbial molecules from the gut into the blood circulation (leaky gut) in the early phase of dengue. The responses against pathogen-associated molecules (PAMPs) from a leaky gut are the possible sources of innate immune responses in dengue, especially during the febrile phase. Notably, the dengue-induced dysbiosis might be due to either a direct enterocyte infection (a reduced mucin and anaerobic microenvironment [23]) or the stress (the selected growth of some bacteria that are able to recognize human stress hormones and norepinephrine [24]). Despite the non-specificity of abdominal pain and vomiting in many causes of fever, dengue cases with GI symptoms, together with an elevation of some specific biomarkers (NETs, FGF-21, or gut microbiome alteration), might indicate severe innate immune responses that should be closely observed. Thus, we hypothesized that (i) abdominal symptoms during the febrile phase in dengue might be correlated with the increased innate immune parameters, especially NETs and FGF-21, and fecal dysbiosis, and (ii) FGF-21 might attenuate inflammation in dengue.

Hence, samples (blood and feces) from cases of dengue with intestinal symptoms (abdominal pain, vomiting, or diarrhea) or without these symptoms during the febrile phase (less than 5 days from the onset of fever) were analyzed. In parallel, several in vitro experiments on enterocytes, macrophages, and neutrophils with endotoxin (a representative PAMP); the dengue virus, and the recombinant FGF-21 were conducted to determine the possible correlation of these molecules.

## 2. Materials and Methods

### 2.1. Enrolled Participants and Study Design

A cross-sectional study was carried out with the approval of the Chulalongkorn University Faculty of Medicine’s Ethics Committee (IRB number 753/64; approval date 1 November 2023), with written informed consent and adherence to the STROBE criteria. The study was conducted between November 2023 and September 2024. Dengue infection was supported by a reverse-transcription polymerase chain reaction (RT–PCR); an NS1 antigen test using an enzyme-linked immunosorbent assay (ELISA) (Platelia, Bio-Rad, Hercules, CA, USA); and dengue immunoglobulin (Ig) M and IgG serology assays (Capture ELISA) (Panbio, Abbott Laboratories, East Brisbane, Australia), in accordance with the World Health Organization’s criteria [25]. Because our main objectives were exploring impacts of innate immunity involved in the early phase of dengue [9], blood samples were collected in patients with fever with or without intestinal symptoms (abdominal pain, vomiting, or diarrhea) within the first 5 days of fever, with the exclusion of the late phase of dengue (dengue hemorrhagic fever). The definition of abdominal pain was the persistent pain in the upper quadrant of the abdomen, and the significant vomiting was defined as more than 3 times a day. Meanwhile, diarrhea was loose stool more than 3 times a day, with stool consistency of 5–7 on the Bristol stool scale (soft blobs with clear-cut edges, fluffy pieces with ragged edges or mushy, and liquid without solid pieces). Notably, sporadic pain or nausea without vomiting or loose stool less than 3 times a day was recorded as no intestinal symptoms. Thus, the inclusion criteria were all adult patients with dengue (age more than 18 years old) who were willing to participate in the study. The exclusion criteria were (i) severe dengue (severe plasma leakage, shock, respiratory distress, severe organ impairment, clinical signs of fluid accumulation, mucosal bleeding, lethargy, and hepatomegaly) [26,27], (ii) end-organ damage [28], and (iii) pregnancy [29]. The dengue infection assessment was based on the 2009 WHO guidelines, and the onset of fever was estimated from the patients’ information [25]. Due to this being a proof-of-concept study, only 20 patients in each group were analyzed.

### 2.2. Blood Sample Analyses

The complete blood count (CBC), CRP, blood urea nitrogen (BUN), serum creatinine (Scr), aspartate transaminase (AST), and ALT were measured at the central laboratory of the King Chulalongkorn Memorial Hospital using a Sysmex XN9203 Analyzer (Kobe, Hyogo, Japan) and a Cobas c502 (Roche Diagnostics, Basel, Switzerland). The serum cytokines (IFN-α, TNF-α, and IL-6) and FGF-21 were measured by enzyme-linked immunosorbent assays (ELISAs) from Invitrogen (Waltham, MA, USA) and Abcam (Cambridge, UK), respectively. The serum endotoxin was determined by an HEK-Blue LPS detection (InvivoGen, San Diego, CA, USA) [30]. The peripheral blood neutrophils were isolated by a Polymorph prep (Alere Technologies AS, Oslo, Norway) with hypotonic lysis buffer (red blood cell decontamination) according to the manufacturer’s instructions before the determination of NETs [31]. The neutrophils resuspended in Roswell Park Memorial Institute media-1640 media (RPMI) with more than 95% neutrophils (Wright’s stains) were further evaluated for NETs by the staining of nuclear morphology using DAPI (4′,6-diamidino-2-phenylindole) color and scored as the percentage of cells with branching from the nuclei [32]. Additionally, NETs formation in the serum was also determined by cell-free DNA (cfDNA) and serum citrullinated histone 3 (CitH3) using a the Quant-iT™ PicoGreen dsDNA Assay Kit (Thermo Fisher Scientific, Waltham, MA, USA) and Cayman Chemical (Ann Arbor, MI, USA), respectively, as previously described [33].

### 2.3. Fecal Microbiome Analysis

A FastPrep-24™ Classic bead beating grinder and lysis system (MP Biomedicals, Santa Ana, CA, USA) and a SPINeasy™ DNA Pro Kit for fecal samples (MP Biomedicals, CA, USA) for metagenomic DNA preparation were used for fecal sample preparation, supported by a DeNovix QFX Fluorometer (DNA quality and quantity). Amplification of the prokaryotic 16S rRNA gene region was performed by a Kinnex^TM^ 16S rRNA kit (Pacific Biosciences, Menlo Park, CA, USA), and 16S RNA amplicons were purified by the SMRTbell cleanup beads (Pacific Biosciences, CA, USA). The quantity and quality of the SMRTbell library were evaluated using a QIAxcel Advanced (Qiagen, Hilden, Germany) and DeNovix QFX Fluorometer (Wilmington, DE, USA), respectively, and the Sequel IIe platform. Sequencing of the SMRTbell library was used following the manufacturer’s protocol. The raw sequences were segmented and demultiplexed into groups based on the Kinnex barcode sequences using SMRT Link software version 13.1 (https://www.pacb.com/smrt-link/, accessed on 8 December 2025) and were processed with the HiFi-16S-Workflow (https://github.com/PacificBiosciences/HiFi-16S-workflow/, accessed on 8 December 2025). The DADA2 pipeline describes microbial diversity and community structures using the unique amplicon sequence variants (ASVs), and the microbial taxa were classified from the Silva version 138.2 reference database. The alpha diversity index (Chao1 richness and Shannon) was computed using DADA2 software version 1.26 using a Kruskal–Wallis test (*p* < 0.05). For the beta diversity, non-metric multidimensional scaling (NMDS) based on Bray–Curtis dissimilarity and a principal coordinate analysis (PCoA) were plotted from Phyloseq data. A permutational multivariate analysis of variance (PERMANOVA) was performed to evaluate the significant differences in beta diversity among groups at *p* < 0.05. The nucleic acid sequences were submitted to an open-access Sequence Read Archive database of NCBI under the accession number PRJNA1377807 (URL https://www.ncbi.nlm.nih.gov/bioproject/?term=PRJNA1377807, submission date 8 December 2025).

### 2.4. In Vitro Experiments

#### 2.4.1. Dengue Virus Propagation and Titer Determination

For dengue virus propagation, dengue virus serotype 2 (DENV-2, strain New Guinea C, NGC) was incubated in Aedes albopictus C6/36 cells from the American Type Culture Collection, Manassas, VA, USA (ATCC CRL-1660), maintained in minimum essential medium (MEM) supplemented with 10% heat-inactivated fetal bovine serum (FBS), 10 mM HEPES buffer, and 1% penicillin–streptomycin (Thermo Fisher Scientific, Waltham, MA, USA) in a humidified 28 °C atmosphere. Then, the C6/36 cells were infected with DENV at a multiplicity of infection (MOI) of 10 [34]; the supernatant was harvested 5 days later using 1500 rpm centrifugation (4 °C, 5 min) and filtered with a 0.22 µm filter. The viral stocks were aliquoted and stored at −80 °C until use. The viral titers were determined by a plaque assay on LLC/MK2 (ATCC^®^ CCL-7) cells maintained in the supplemented MEM (Thermo Fisher Scientific, Waltham, MA, USA) and were seeded at a density of 5 × 10^4^ cells/well in 24-well plates at 37 °C with 5% CO2 until 70–80% confluence was achieved. The infected cells were washed with phosphate buffer solution (PBS) and overlaid with 1 mL supplemented MEM (1% FBS, 10 mM HEPES, 1% penicillin–streptomycin, and 0.8% gum tragacanthin) (Sigma-Aldrich^®^, St. Louis, MO, USA) for 5–10 days and subsequently fixed and stained with 10% formaldehyde solution (Carlo Erba^®^, Milan, Italy), 5% isopropanol (Merck^®^, Darmstadt, Germany), and 1% crystal violet (Merck^®^, Darmstadt, Germany) for 1 h. The number of plaque-forming units per milliliter (PFU/mL) was determined manually.

#### 2.4.2. Hepatocyte Culture, Stimulation, and Starvation Protocol

HepG2 (ATCC HB-8065) (Manassas, VA, USA) was maintained in supplemented Dulbecco’s modified Eagle medium (DMEM) consisting of 5.5 mM glucose at 37 °C under 5% CO2 and sub-cultured before use in the experiments. The cells, at 1 × 10^6^ cells/well were then incubated with lipopolysaccharide (LPS) from *E. coli* O26: B6 (Sigma-Aldrich, St. Louis, MO, USA) (100 ng/mL), or DENV at MOI 10 or the combination of both factors for 48 h before determining the supernatant cytokines by ELISAs from Quantikine Immunoassay (R & D Systems, Minneapolis, MN, USA) and from Abcam (Cambridge, UK). Additionally, a starvation protocol using a medium with no glucose (glucose deprivation or glucose-free medium) was performed as a positive control condition for FGF-21 induction [19]. The gene expression of fibroblast growth factor receptor 1 (*FGFR1*) was selected by a quantitative real-time polymerase chain reaction (PCR) using the RNA extracted from cell pellets by a FarvoPrep RNA mini kit (Farvogen, Vienna, Australia). The amounts of RNA were quantified using a NanoDrop OneC Microvolume UV-Vis Spectrophotometer (Thermo Fisher Scientific, Wilmington, DE, USA), and the cDNA was synthesized using a cDNA reverse transcription kit (Applied Biosystems, Warrington, UK). The quantitative PCR was performed using an SYBR^®^ Green PCR Master Mix and an Applied Biosystems QuantStudio 6 Flex Real-Time PCR System (Applied Biosystems, Thermo Fisher Scientific, Waltham, MA, USA). The results are shown in terms of the relative quantification of the comparative threshold method (delta-delta Ct; 2^−ΔΔCt^) as normalized by β-actin (an endogenous housekeeping gene). The primers for human genes were derived from previous publications [30] and are shown in Table 1.

#### 2.4.3. Macrophage Differentiation, Primary Human Neutrophil Isolation, and Stimulations

Impacts of LPS and DENV were also tested in THP-1-differentiated macrophages. For this, a THP-1 cell line (ATCC TIB-202, Manassas, VA, USA) was cultured in RPMI 1640 supplemented with 10% heat-inactivated fetal bovine serum, 100 U/mL penicillin, 100 µg/mL streptomycin, and 50 µM 2-mercaptoethanol (Invitrogen, Waltham, MA, USA). Subsequently, macrophages, at 1 × 10^6^ cells/well were incubated with LPS with or without DENV as mentioned above for 24 h before the determination of supernatant cytokines using an ELISA (Quantikine Immunoassay) (R&D Systems, Minnesota, MN, USA) and gene expression by PCR. In parallel, the isolated neutrophils from peripheral blood, at 5 × 10^5^ cells/well, were activated by LPS or DENV or their combination for 6 h before determination of supernatant cytokines and *FGFR1* expression, as mentioned above. For NETs formation, the supernatant CitH3 and cfDNA were measured by an ELISA from Cayman Chemical (Ann Arbor, MI, USA) and a Quant-iT™ PicoGreen ELISA Assay (Thermo Scientific, Waltham, MA, USA), respectively, together with DAPI-stained nuclear morphology and the expression of protein arginine deiminase 4 (*PAD4*) gene by PCR, as described above.

#### 2.4.4. Recombinant FGF-21 Co-Incubation Assays

To explore the possible action of FGF-21, Caco-2 or macrophages or neutrophils were activated by LPS (100 ng/mL) plus DENV (MOI 10) with or without recombinant human FGF-21 (Sigma-Aldrich, Burlington, MA, USA) at 100 ng/mL before the determination of supernatant cytokines (Quantikine Immunoassay, R & D Systems, Minnesota, MN, USA). Additionally, NETs were determined using immunofluorescent staining by anti-neutrophil elastase (NE; ab68672) and anti-myeloperoxidase (MPO; ab25989) before visual analysis using a confocal fluorescent microscope (ZEISS LSM 800, Carl Zeiss, Jena, Germany), as previously published [35].

### 2.5. Statistical Analysis

The mean ± standard error of the mean (SEM) was presented using the student’s T test or one-way analysis of variance (ANOVA) followed by Tukey’s analysis for two and multiple group comparisons, respectively. All statistical analyses were performed with GraphPad Prism version 10.0 software (La Jolla, CA, USA), and a *p*-value of <0.05 was considered statistically significant.

## 3. Results

### 3.1. Higher NETs, FGF-21, and Fecal Proteobacteria in Dengue with Febrile Intestinal Symptoms (Abdominal Pain, Vomiting, or Diarrhea)

**Figure 1:**
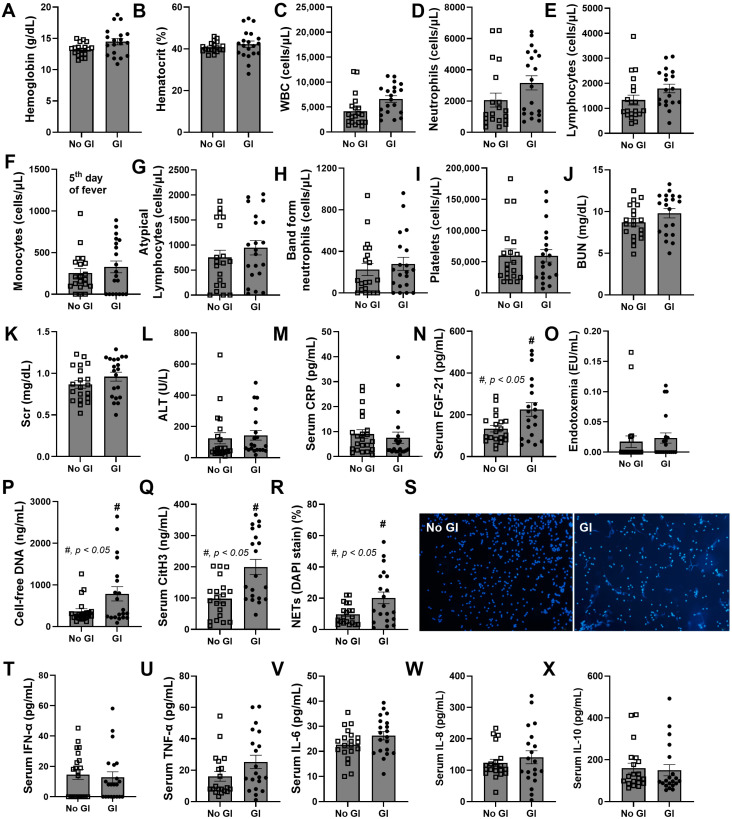
Characteristics of dengue without intestinal symptoms (no GI) (*n* = 20) and dengue with intestinal symptoms (GI) (*n* = 20), as indicated by complete blood count (CBC) (**A**–**I**); renal function (blood urea nitrogen (BUN) and serum creatinine (Scr)) (**J**,**K**); liver responses, including alanine transaminase (ALT), C-reactive protein (CRP), and fibroblast growth factor 21 (FGF-21) (**L**–**N**); endotoxemia (**O**); parameters of neutrophil extracellular traps, including cell-free DNA, citrullinated histone 3 (CitH3), and nuclear morphology stained by DAPI (4′,6-diamidino-2-phenylindole) with representative pictures (**P**–**S**); and serum cytokines (IFN-α, TNF-α, IL-6, IL-8, and IL-10) (**T**–**X**). #, *p* < 0.05 versus no GI control.

Because neutrophils might be responsible for the early responses in dengue with intestinal symptoms, samples were collected before the 5th day of fever. A diagnosis of dengue was supported by NS1 and PCR, while the prominent serotype was DENV2 (Table 2, dengue serotype). In the febrile dengue without gastrointestinal symptoms (the no GI group), all cases had fever with myalgia, 15 cases complained of anorexia (unable to eat), and three cases had nausea, but there was no vomiting or diarrhea in this group (Table 2, symptoms). In the febrile dengue with gastrointestinal symptoms (the GI group), all 20 cases had abdominal pain and anorexia, 18 cases had vomiting (1–3 times per day), and seven patients had diarrhea (positive for abdominal pain in all cases but no vomiting in this group) (Table 2, symptoms). Although the inclusion criteria specified recruited only the cases with upper quadrant abdominal pain, there were no cases with pain in other quadrants of the abdomen identified during the recruitment. Among the 20 cases with abdominal pain, 15 cases reported right upper quadrant pain, three cases with pain at the epigastrium, and two cases with the left upper quadrant pain. Despite the association between abdominal pain and liver swelling, hepatomegaly in the febrile phase was demonstrated only in four out of 20 patients with abdominal pain (Table 2, symptoms). The diarrhea was reported on the third to fourth days after fever onset, which lasted only for one day without signs of dehydration. The stool consistency in all cases was categorized as the Bristol stool type 6 (soft stool with ragged edges, not entirely liquid). In comparison with the no GI group, the CBC for the GI group indicated increased atypical lymphocytes and band-form neutrophils with thrombocytopenia without other white blood cell (WBC) abnormalities (Figure 1A–I). Notably, the hemoconcentration (a rise in hematocrit of more than 20% from the baseline) could not be determined due to the cross-sectional analysis. There was no renal injury, as indicated by the BUN and Scr, in either groups (Figure 1J,K). For the liver responses (ALT, CRP, and FGF-21), only the FGF-21 in the GI group was higher (Figure 1L–N) than in the no GI group. Endotoxemia was similar in both groups, implying a similar severity of leaky gut (Figure 1O). On the other hand, all parameters of NETs formation in the GI group, as indicated by the cfDNA, CitH3, and nuclear morphology using DAPI staining, in the GI group were higher than in the no GI group (Figure 1P–S), while the serum cytokine panels (IFN-α, TNF-α, IL-6, IL-8, and IL-10) were similar between groups (Figure 1T–X).

**Figure 2:**
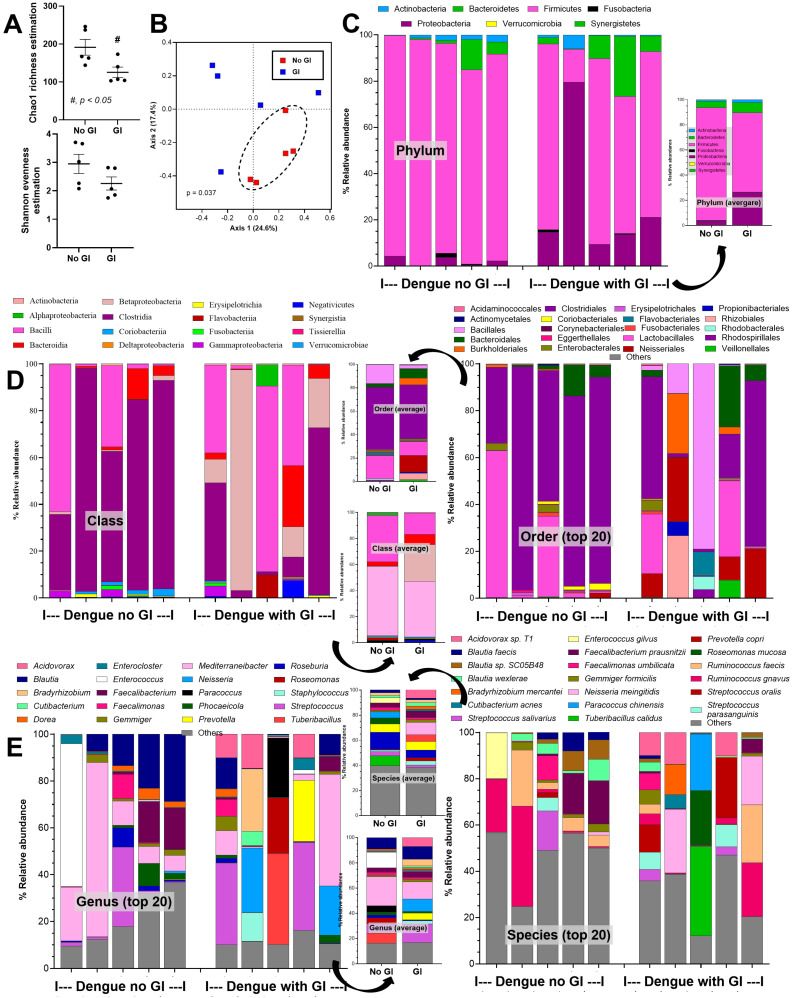
Fecal microbiome analysis of dengue without intestinal symptoms (no GI) (*n* = 5) and dengue with intestinal symptoms (GI) (*n* = 5), as indicated by alpha diversity (Chao-1 and Shannon) (**A**); beta diversity (Bray–Curtis dissimilarity analysis) (**B**); and bacterial abundance, with the average values for phylum, class, order, genus, and species (**C**–**E**) shown. #, *p* < 0.05 versus no GI control.

**Figure 3:**
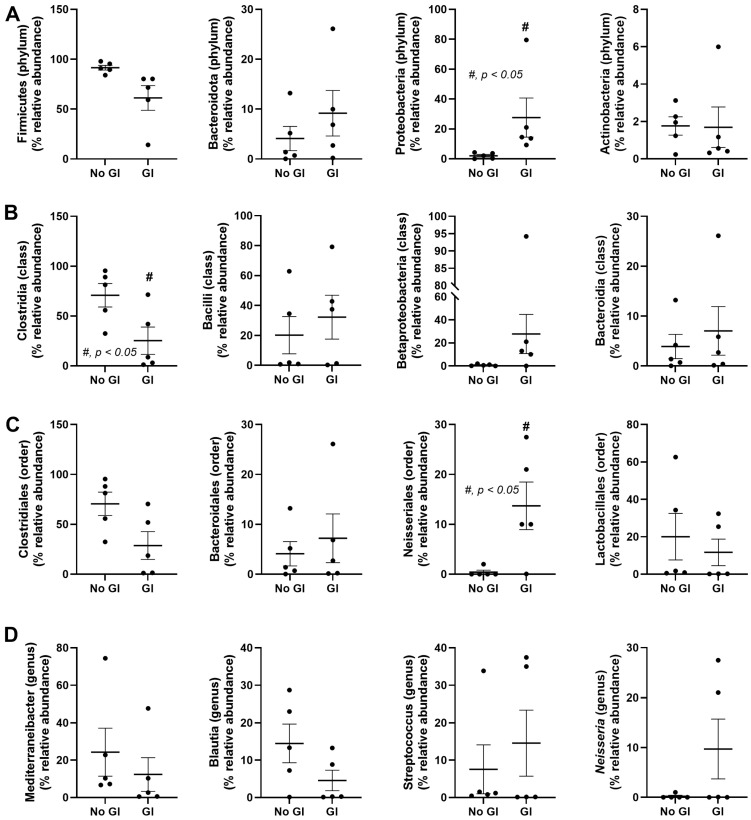
Fecal microbiome analysis of dengue without intestinal symptoms (no GI) (*n* = 5) and dengue with intestinal symptoms (GI) (*n* = 5), as indicated by some of the graphs of bacterial abundance in phylum (**A**), class (**B**), order (**C**), and genus (**D**). #, *p* < 0.05 versus no GI control.

Additionally, fecal microbiome analyses were performed for the five randomly selected patients per group, and two patients in the GI group had diarrhea. For the alpha diversity, there was a lower Chao-1 (a richness score) in the GI group compared with the no GI group without a difference in the Shannon score (an evenness score) (Figure 2A). Meanwhile, the beta diversity using a Bray-Curtis dissimilarity analysis demonstrated a separation between groups (Figure 2B). Notably, the phylogenetical-based analysis concerning the unique evolutionary branch length shared between two communities, referred to as the UniFrac (weighted and unweighted) analysis, is less appropriate for gut microbiota due to the high taxonomic homogeneity in the gut (primarily Firmicutes and Bacteroidetes) different from microbiome analysis of environmental sources [36]. A non-phylogenetic Bray–Curtis analysis determines bacterial abundance better than an unweighted UniFrac that relies on the relative abundance (proportional data) [37]. However, there was only a subtle difference in fecal bacterial abundance (phylum, class, order, genus, and species levels) between groups (Figure 2C–E). In the statistical analysis, there was higher Proteobacteria (phylum level), lower Clostridia (class level), and higher Neisseriales (order level), without a difference in the genus and species levels in the GI group compared with the no GI group (Figure 3A–D). Despite the significantly elevated Proteobacteria in the GI group compared to the no GI group (Figure 3A), there were only two samples with an abundance of Proteobacteria that was high enough to be detectable in the top 20 species levels (*Neisseria meningitidis*), resulting in the non-significant difference between groups (Figure 3D). From the average relative abundance at the phylum level (Figure 2C, right side), the predominant bacteria in the no GI and GI groups were Firmicutes and Firmicutes with Proteobacteria, respectively (Figure 2C).

### 3.2. Endotoxin and Dengue Upregulate FGFR1 to Enhance Anti-Inflammatory FGF-21

**Figure 4:**
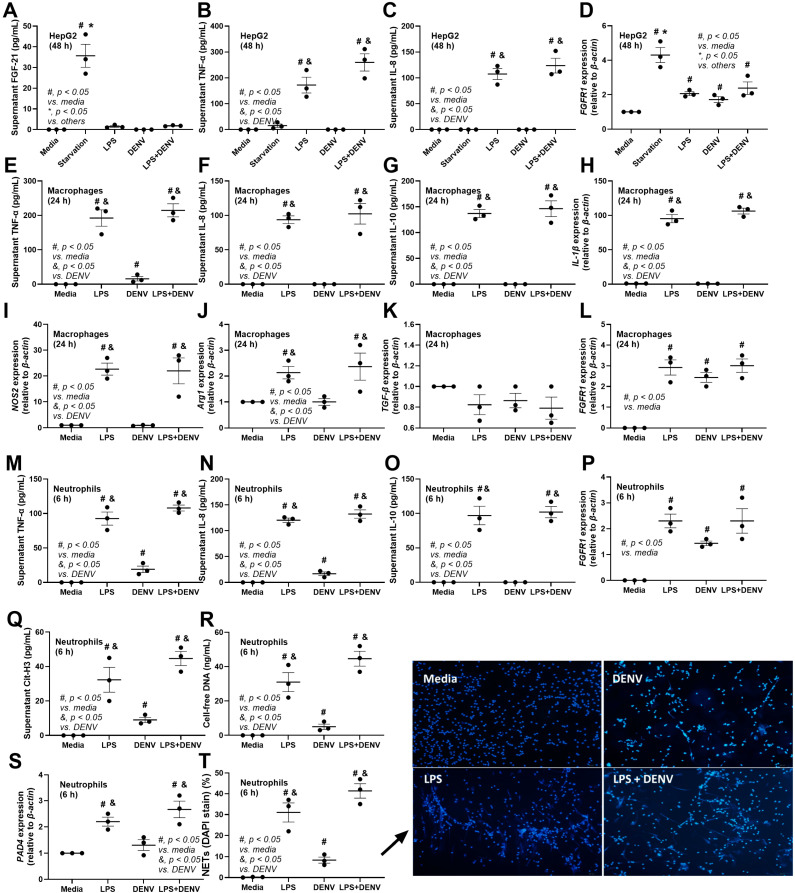
Characteristics of a human liver cell line (HepG2 cells) after stimulation by control media (media), starvation protocol, lipopolysaccharide (LPS), and dengue virus (DENV), as indicated by supernatant fibroblast growth factor 21 (FGF-21), cytokines (TNF-α and IL-8), and expression of FGF receptor 1 (*FGFR1*) gene (**A**–**D**). Characteristics of THP-1-derived macrophages after stimulation by media, LPS, DENV, and LPS plus DENV (LPS + DENV), as indicated by supernatant cytokines (TNF-α, IL-8, and IL-10) with the expression of M1 polarization genes (*IL-1β* and *NOS2*), M2 polarization genes (TGF-β and Arg-1), and *FGFR1* expression (**E**–**L**) shown. Characteristics of isolated neutrophils from healthy volunteers after stimulation by media, LPS, DENV, and LPS + DENV, as indicated by supernatant cytokines (TNF-α, IL-8, and IL-10); *FGFR1* expression; and parameters of neutrophil extracellular traps (NETs), including citrullinated histone 3 (CitH3), cell-free DNA, peptidyl arginine deiminase 4 (*PAD4*) expression, and nuclear morphology staining by DAPI (4′,6-diamidino-2-phenylindole), with representative pictures (**M**–**T**) shown. Data are derived from 3 isolated experiments. #, *p* < 0.05 versus media; *, *p* < 0.05 vs. others; &, *p* < 0.05.

**Figure 5:**
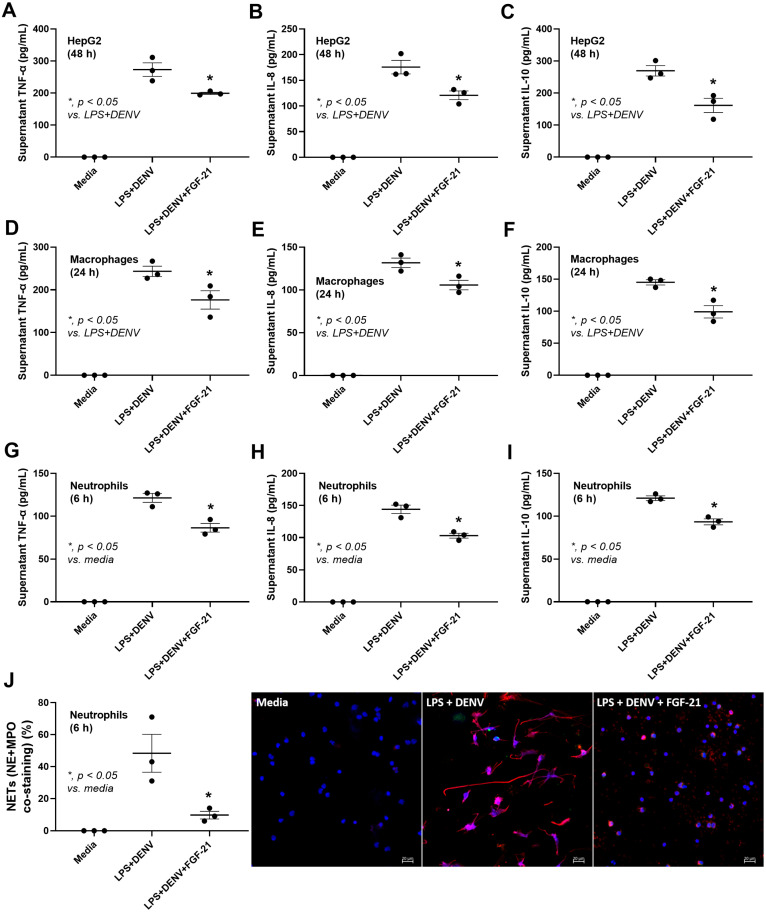
Characteristics of a human liver cell line (HepG2 cells), THP-1-derived macrophages, and isolated neutrophils from volunteers after the combination of LPS with DENV (LPS + DENV) or the activation with recombinant FGF-21 (LPS + DENV + FGF-21), as indicated by supernatant cytokines (TNF-α, IL-8, and IL-10) (**A**–**I**) and NETs using co-staining with the neutrophils elastase (NE) and myeloperoxidase (MPO), with representative pictures (**J**) shown. Data are derived from 3 isolated experiments. *, *p* < 0.05 vs. LPS + DENV.

Because (i) FGF-21 is mainly produced by the liver [10] and (ii) endotoxin and dengue viral particles are microbial molecules that might induce an immune response during infection, the in vitro experiments using endotoxin (lipopolysaccharide or LPS), dengue (DENV), and FGF-21 were used to explore the possible connection. In hepatocytes, only cell starvation (glucose deprivation), but not LPS, DENV, or their combination (LPS+DENV), stimulated FGF-21 excretion (Figure 4A). While LPS and LPS+DENV similarly elevated supernatant cytokines (TNF-α and IL-8) (Figure 4B,C), starvation potently upregulated the FGF receptor 1 gene (*FGFR1*) (Figure 4D), but not inflammatory cytokines (Figure 4B,C). Notably, LPS, DENV, and LPS+DENV also upregulated *FGFR1* (Figure 4D), implying the role of microbial molecules in facilitating the FGF-21 effect, but they did not directly induce FGF-21 excretion from the livers. In the THP-1-differentiated macrophages, LPS and LPS+DENV similarly enhanced inflammatory cytokines (TNF-α, IL8, and IL-10) (Figure 4E–G), M1 pro-inflammatory parameters (*IL-1β* and *NOS2* expression) (Figure 4H,I), and M2 anti-inflammatory markers (*Arg-1* expression but not *TGF-β*) (Figure 4J,K); and DENV induced only mild supernatant TNF-α elevation (Figure 4E). However, LPS, DENV, and LPS+DENV similarly upregulated *FGFR1* (Figure 4L). In neutrophils, LPS and LPS+DENV similarly elevated supernatant cytokines (TNF-α, IL-8, and IL-10) (Figure 4M–O), *FGFR1*(Figure 4P), and NETs, as determined by CitH3(Figure 4Q), cfDNA (Figure 4R), peptidyl arginine deiminase 4 (*PAD4*) expression (Figure 4S), and DAPI nuclear morphology (Figure 4T). In the neutrophils, DENV increased cytokines (TNF-α and IL-8 but not IL-10) (Figure 4M–O) and induced NETs (CitH3, cfDNA, and DAPI nuclear morphology but not *PAD4* expression) at a lower intensity than in the LPS and LPS+DENV activations (Figure 4Q–T). Meanwhile, the upregulated *FGFR1* was similar to that produced by LPS and LPS+DENV in the neutrophils (Figure 4P). To explore the possible function of FGF-21, the human recombinant FGF-21 was incubated with LPS+DENV-activated cells (HepG2, macrophages, and isolated neutrophils). As a result, FGF-21 reduced the supernatant cytokines (TNF-α, IL-6, and IL-10) in hepatocytes (Figure 5A–C), macrophages (Figure 5D–F), and neutrophils (Figure 5G–I). In parallel, FGF-21 also decreased the NETs, as determined by staining with NE and MPO (Figure 5J), indicating a role of innate immunity in controlling inflammation in dengue.

## 4. Discussion

The elevation of NETs and serum FGF-21 in patients with GI symptoms, especially constant abdominal pain in the upper abdomen, during the febrile phase of dengue (less than 5 days of fever) indicated severe inflammatory responses and a possible requirement for intensive monitoring.

### 4.1. Constant Abdominal Pain During Dengue Febrile Phase Is an Important Warning Sign

Constant abdominal pain is an earlier warning sign than fluid accumulation, mucosal bleeding, and lethargy in dengue that occurs during the non-febrile critical phases [38]. Pain Not only during the non-febrile critical phase, but also constant abdominal pain, especially in the upper quadrant, even during the febrile phase of dengue (less than 5 days of fever), can also be a concern for developing the severe disease. While the non-specific sporadic cramping pain (increased intestinal peristalsis) is not concerning [39], constant abdominal pain in any abdominal locations in patients with dengue in any phase of the disease is important. Here, there were no cases with vomiting alone or diarrhea alone without constant abdominal pain, implying that pain is the most important warning symptom. Because most diarrhea in dengue is reported without abdominal pain [40,41], diarrhea is not a warning sign in dengue. Although we did not have any diarrhea cases without abdominal pain during the observation period, a previous publication reported diarrhea with less predominant abdominal pain in 13–37% and up to 33% of cases in adults and in children, respectively, during the febrile phase (2–5 days), for a duration of 2 to 4 days with a very low severity of both dengue and diarrhea [42]. The severity of dengue-induced diarrhea in some adult patients is too low to be recognized as a manifestation [43]. Hence, we concluded that the constant abdominal pain during the febrile phase, but not diarrhea, should be a concern. Likewise, the constant abdominal pain is also a stronger sign than vomiting, which can be caused by several non-specific causes. Despite the benefit of constant abdominal pain as a warning sign, the additional objective confirmatory laboratories might be helpful for clinicians.

### 4.2. Enterocyte Infection with Dengue and Its Clinical Importance

Although there is no report on the direct identification of the dengue virus from an intestinal tissue biopsy due to the possible bleeding risk, DENV infects human enterocytes through both apical and basolateral surfaces via clathrin-mediated endocytosis [44,45]. These data indirectly support the infectivity of DENV in the intestines. In immunocompromised mice (such as interferon gamma receptor-deficient models), the DENV-induced gut injury (prominent edema, villus blunting, exfoliation, crypt abscesses, and the thinning of the mucus layer) was demonstrated before liver and spleen injuries [21]. Hence, the data in mice support direct enterocyte infection by DENV. Nevertheless, the correlation between the GI symptoms and enterocyte infectivity of dengue in humans is still uncertain and can not be concluded from our data. Based on the dengue-induced gut dysbiosis [46], our data suggested a different fecal dysbiosis in patients with and without GI symptoms (pain and diarrhea). The higher abundance of Proteobacteria (a group of Gram-negative bacteria with leaky gut-inducing properties) in patients with GI symptoms implies more prominent enterocyte impacts than in the no GI cases. It is possible that DENV might infect the intestinal cells in all cases but demonstrate GI symptoms, especially diarrhea, only in the susceptible cases, or DENV might infect enterocytes only in the cases with high-abundance viremia. More studies would be interesting. Here, we demonstrated the possible clinical importance of GI symptoms during the febrile phase to predict dengue disease severity. However, the clinical importance of enterocyte infectivity by DENV in humans is still inconclusive.

### 4.3. Higher Serum FGF-21 and NET-Associated Parameters in Patients with Gastrointestinal Symptoms During the Febrile Phase of Dengue

Due to the rapid response of innate immunity, a good confirmatory laboratory test in addition to the constant abdominal pain in dengue might be an indication of the responses from macrophages, neutrophils, and the liver. Although macrophages are the main innate immune cells that contribute to elevated cytokines in dengue [47], there are no solid representative cytokines for the disease’s severity determination [48,49]. Here, cytokines could not differentiate dengue with and without GI symptoms, while NETs, an important neutrophil response, were higher in dengue with GI symptoms. Among several NETs-associated parameters, CitH3 and cfDNA can easily be detected in serum, while DAPI staining of the isolated neutrophil portion is too complicated for routine clinical practice. Although cfDNA was previously mentioned as a dengue severity biomarker [50], cfDNA originates not only from neutrophils but also from other damaged cells [51], and elevated cfDNA is not a good representative of neutrophil response. Thus, CitH3 might be a more neutrophil-correlated parameter for supporting the prominent inflammation in the febrile phase with constant abdominal pain. Also, acute-phase proteins (APPs), the circulating proteins produced by the liver in response to inflammation, were mentioned as a severity biomarker of dengue [52]. Here, CRP (a well-known APP) and ALT could not differentiate dengue with and without GI, but FGF-21 (a molecule mainly produced during liver stress) could differentiate both groups. On the other hand, gut dysbiosis, as indicated by increased Proteobacteria (mostly harmful bacteria in this group) with reduced Clostridiales (several beneficial bacteria in this group), was another parameter that could differentiate dengue with and without GI symptoms; however, the inconvenient fecal collection is not suitable for a routine clinical practice. Notably, the dengue-induced dysbiosis is caused by cytokine activation and enterocyte stress from viral infection that might reduce mucin production and anaerobic conditions in the gut, allowing for overgrowth of harmful bacteria (e.g., Gram-negative aerobes in the Proteobacterial phylum) [53]. Hence, CitH3 or FGF-21 might be good parameters to use as a support parameter for febrile abdominal pain in dengue. More studies would be interesting.

### 4.4. The Anti-Inflammatory Properties of FGF-21 for Liver Stress in Dengue

**Figure 6:**
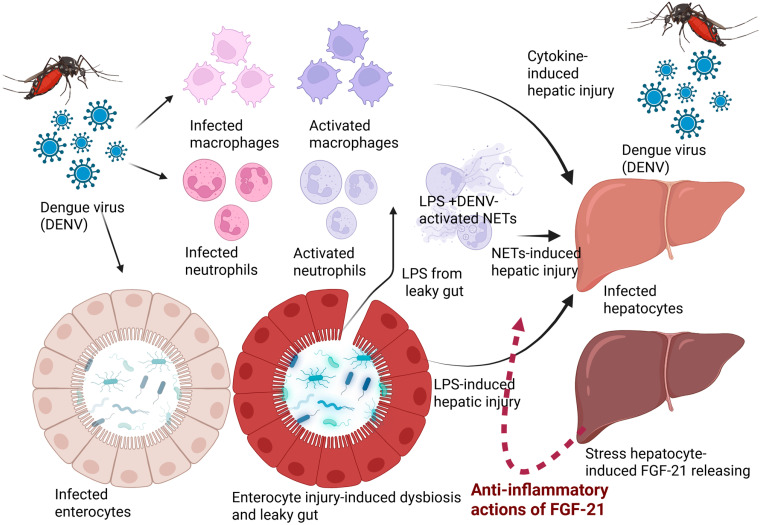
A schema of the possible hypothesis regarding the innate immune responses and FGF-21 production during dengue infection is presented. In dengue with warning signs, there might be a direct DENV infection in several cells, including monocytes/macrophages (major cytokine-producing cells) [54], neutrophils (NETs formation) [55], enterocytes (causing gut dysbiosis, leaky gut, and endotoxemia) [56], and hepatocytes (acute phase protein production) [57,58], which induces the production of fibroblast growth factor 21 (FGF-21) from the stressed liver in order to attenuate inflammation [61,62]. Further studies on innate immune responses in dengue would be interesting. BioRender (URL https://BioRender.com) was used to create and illustrate the figure illustration.

Because of the viremia in the febrile phase of dengue, and its well-known inflammatory activation by LPS, LPS and the dengue virus (DENV) were used to test the responses of several cells. Although the major sources of FGF-21 are the liver, adipose tissue, and pancreas [18], hepatocytes are the well-known source of FGF-21. Here, only the starvation protocol, but not LPS or DENV, elevated the FGF-21 level, supporting the more prominent impact of liver stress than that of microbial molecules on FGF-21 production. Meanwhile, only LPS, but not DENV, facilitated hepatocyte cytokine production, indicating the more prominent pro-inflammatory activity of the bacterial molecule compared with the viral molecule. On the other hand, the microbial molecules (LPS and LPS+DENV) induced proinflammation in both macrophages (cytokine production) and neutrophils (cytokines and NETs). Despite the lower inflammatory activation of DENV in comparison with LPS, both LPS and DENV similarly upregulated the *FGFR1* gene in all cells, which might have enhanced the anti-inflammatory function of FGF-21. Here, the anti-inflammatory effects of FGF-21 were demonstrated by reduced cytokine production in all cells (hepatocytes, macrophages, and neutrophils), with the attenuation of NET formation. Thus, the microbial molecules induced inflammation and upregulated *FGFR1* in several cells, possibly for a counteracting response, enhancing the anti-inflammation effect of FGF-21 produced from the stressed livers during dengue infection. Due to the possible direct dengue viral infection of several cells (macrophages and neutrophils) [54,55] and organs (intestines and livers) [56,57,58] during viremia, the responses to infection through cytokines, NETs, and dysbiosis might be attenuated by FGF-21 (Figure 6). For clinical applications, both CitH3 and/or FGF-21 in serum may be good inflammatory biomarkers to support the diagnosis of severe dengue in patients with febrile abdominal pain. The use of each or both biomarkers might alert the physicians to severe dengue. More studies are warranted.

### 4.5. Limitations and Future Directions

Several limitations should be mentioned. First, there are a limited number of patients with hematocrit data at the baseline or during the convalescence phase to determine the hemoconcentration. A higher number of patients with more time points of blood collection will be needed. Second, only DENV2, but not DENV1, 3, or 4, was used in vitro due to the clearer viral plaque that forms during the in vitro preparation, resulting in the frequent use of DENV2 in several previous publications [59]. The use of the other DENV serotypes in vitro would be interesting. Third, the infectivity of DENV in human enterocytes was not determined. The identification of DENV from enterocytes using the non-invasive procedures, such as the use of shading (exfoliative) enterocytes in fecal contents of patients [60], would be very interesting.

## 5. Conclusions

Constant abdominal pain during the febrile phase of dengue serves as a clinical indicator of prominent innate immune activation, characterized by elevated NETs, serum FGF-21, and gut dysbiosis. Our findings indicate that liver-derived FGF-21 is produced in response to infection-induced stress and functions as a crucial anti-inflammatory mediator to attenuate systemic inflammation and NETs. Therefore, monitoring serum CitH3 and FGF-21 provides a robust strategy for the early identification of high-risk patients, and the use of recombinant FGF-21 might be clinically interesting. Also, the liver–neutrophil axis and the possible role of FGF-21 are highlighted in the innate immunity of dengue pathogenesis.

## Figures and Tables

**Table 1:** Primers used in the study.

Biological Function	Name	Forward Primer	Reverse Primer
Pro-inflammatory markers	Interleukin 1-beta (*IL-1β*)	5’-CCACAGACCTTCCAGGAGAATG-3’	5’-GTGCAGTTCAGTGATCGTACAGG-3’
	Inducible nitric oxide synthase (*NOS-2*)	5’-CAGCGGGATGACTTTCCAAG-3’	5’-AGGCAAGATTTGGACCTGCA-3’
Anti-inflammatory markers	Arginase-1 (*Arg-1*)	5’-TGGACAGACTAGGAATTGGCA-3’	5’-CCAGTCCGTCAACATCAAAACT-3’
	Transforming growth factor beta (*TGF-β*)	5′-TACCTGAACCCGTGTTGCTCTC-3′	5′-GTTGCTGAGGTATCGCCAGGAA-3′
NETosis marker	Peptidyl arginine deiminase 4 (*PAD4*)	5′-CGAAGACCCCCAAGGACT-3′	5′-AGGACAGTTTGCCCCGTG-3′
*Housekeeping gene*	*β-actin*	5’-CCTGGCACCCAGCACAAT-3’	5’-GCCGATCCACACGGAGTACT-3’

**Table 2:** The characteristics of participants.

Data	No GI (*n* = 20)	GI (*n* = 20)
Age (year, mean ± SE *)	27 ± 6	30 ± 8
Female, *n* (%)	9 (45)	10 (50)
NS1 positive, *n* (%)	20 (100)	20 (100)
PCR positive, *n* (%)	20 (100)	20 (100)
Dengue serotype, *n* (%)		
Serotype DENV-1	5 (25)	2 (10)
Serotype DENV-2	15 (75)	17 (85)
Serotype DENV-3	0	1 (5)
Serotype DENV-4	0	1 (5)
Comorbidity, *n* (%)		
Diabetes	0	1 (5)
Hypertension	0	3 (15)
Symptoms, *n* (%)		
Fever	20 (100)	20 (100)
Anorexia	15 (75)	20 (100)
Nausea	3 (15)	20 (100)
Vomiting	0 (0)	18 (90)
Abdominal pain	0 (0)	16 (80)
Hepatomegaly	0 (0)	4 (20)
Diarrhea	0 (0)	7 (35)
Myalgia	20 (100)	20 (100)
Rash	0 (0)	0 (0)
Mucosal bleeding	0 (0)	0 (0)
Fluid accumulation	0 (0)	0 (0)

*, standard error; No GI, dengue without intestinal symptoms; GI, dengue with intestinal symptoms (abdominal pain or vomiting or diarrhea).

## Data Availability

The data are contained within the article.

## Abbreviations

The following abbreviations are used in this manuscript:

GI	Gastrointestinal
NETs	Neutrophil extracellular traps
FGF-21	Fibroblast growth factor 21
LPS	Lipopolysaccharide
DENV	Dengue virus
CitH3	Citrullinated histone 3
